# Lipids, cholesterols, statins and liver cancer: a Mendelian randomization study

**DOI:** 10.3389/fonc.2023.1251873

**Published:** 2023-09-06

**Authors:** Zicheng Liang, Zhen Zhang, Xiaoning Tan, Puhua Zeng

**Affiliations:** ^1^ Graduate School, Hunan University of Traditional Chinese Medicine, Changsha, China; ^2^ Department of Oncology, Affiliated Hospital of Hunan Academy of Chinese Medicine, Changsha, China

**Keywords:** Mendelian randomization, lipids, cholesterols, statins, liver cancer

## Abstract

**Aim:**

To investigate the causal relationship of serum lipid indicators and lipid-lowering drugs with the risk of liver cancer using Mendelian randomization study.

**Methods:**

A two-sample Mendelian randomization (TSMR) study was performed to investigate the causal relationship between serum levels of lipid indicators and liver cancer, including low-density lipoprotein cholesterol (LDL-c), high-density lipoprotein cholesterol (HDL-c), triglycerides (TG), total cholesterol (TC), Apolipoprotein B (ApoB), and Apolipoprotein A1 (ApoA1).Furthermore, instrumental variable weighted regression (IVW) and summary data-based MR (SMR) analyses were performed to investigate the causal effects of lipid-lowering drugs, including statins and PCSK9 inhibitors, on the risk of liver cancer.

**Results:**

Serum LDL-c and serum TC levels showed negatively associated with liver cancer (n = 22 SNPs, OR = 0.363, 95% CI = 0.231 - 0.570; p = 1.070E-5) (n = 83 SNPs; OR = 0.627, 95% CI = 0.413-0.952; p = 0.028). However, serum levels of TG, HDL-c, and ApoA1 did not show any significant correlation with liver cancer. In the drug target MR (DMR) analyses, HMGCR–mediated level of LDL-c showed an inverse relationship with the risk of liver cancer in the IVW-MR analysis (n = 5 SNPs, OR = 0.201, 95% CI = 0.064 - 0.631; p = 5.95E-03) and SMR analysis (n = 20 SNPs, OR = 0.245, 95% CI = 0.065 - 0.926; p = 0.038) However, PCSK9 did not show any significant association with liver cancer based on both the IVW-MR and SMR analyses.

**Conclusion:**

Our results demonstrated that reduced levels of LDL-c and TC were associated with an increased risk of liver cancer. Furthermore, lipid-lowering drugs targeting HMGCR such as statins were associated with increased risk of liver cancer.

## 1 Introduction

Liver cancer is one of the most common malignancies with high incidence and mortality rates. The rapid rise in metabolic diseases has contributed significantly to the increased incidence of liver cancer globally (1). The number of new liver cancer cases are estimated to increase by 55% between 2020-2040; moreover, 1.4 million new liver cancer diagnose and 1.3 million deaths related with liver cancer are predicted by 2040 (2).

Metabolic reprogramming is one of the major hallmarks of liver cancer and includes significant changes in lipid metabolism (3). Aberrant changes in the gene expression levels of genes encoding enzymes regulating lipid metabolism are linked with molecules that modulate carcinogenic signaling pathways (4). The serum levels of low-density-lipoprotein cholesterol (LDL-c), high-density-lipoprotein cholesterol (HDL-c), triglycerides (TG), total cholesterol (TC), Apolipoprotein B (ApoB), and Apolipoprotein A1 (ApoA1) are associated with several human diseases included cancers and are routinely used in clinical diagnosis. For example, the oxidized form of LDL-c (ox-LDL), a carrier protein for cholesterol, is associated with liver steatosis, inflammation, and fibrosis (5). LDL-c is also a prognostic indicator of colorectal cancer and breast cancer (6, 7). Low levels of HDL-c are associated with non-alcoholic fatty liver disease (NAFLD), a risk factor for liver cancer (8). However, the mechanistic link between reduced levels of HDL-c and increased risk of liver cancer is not known. A randomized cohort study of European subjects showed a positive linear relationship between triglycerides and the incidence of liver cancer in male patients (9). Ma X et al. performed a large prospective cohort study in Chinese population and showed that low serum levels of total cholesterol (TC) were associated with an elevated risk of liver cancer (10). ApoB is a major protein component of LDL-c. Low ApoB activity is associated with the upregulation of oncogenic factors such as HGF and MTIF, downregulation of tumor suppressor factors such as TP53 and PTEN, and poor outcomes of patients with hepatocellular carcinoma (11). ApoA1 is a major protein component of HDL-c and plays a critical role in reverse cholesterol transport. ApoA1 is a promising diagnostic indicator of hepatitis B virus-related hepatocellular carcinoma (12). Lipid-lowering drugs such as statins significantly reduce the levels of LDL-c and are associated with reduced risk of hepatocellular carcinoma (13). HMGCR and PCSK9 are the target proteins of drugs that reduce the levels of LDL-c by rewiring lipid metabolism. However, the relationship between these lipid metabolic enzymes and the risk of liver cancer remains to be explored.

Mendelian randomization (MR) is an analytic method used to determine the causal effects of the exposure variables on the outcomes. MR studies are beneficial because they can overcome the limitations of traditional methods in estimating the causal association through observational studies, including confounding and survival biases (14). Univariable MR is used to analyze the relationship between a specific exposure and the outcome after accounting for the potential confounding factors. Drug target Mendelian randomization (DMR) is a valuable method for investigating the effects of biomarkers related to specific drug targets (for example, levels of blood lipids) on the treatment outcomes. DMR is used to determine adverse drug reactions. In this study, we performed univariable MR analyses to determine the causal effects of individual serum lipid indicators on the incidence of liver cancer. Furthermore, we performed a DMR analysis to assess the causal effects of two LDL-c lowering drugs that target HMGCR and PCSK9, respectively, on the risk of liver cancer.

## 2 Materials and methods

### 2.1 Study design

**Figure 1:**
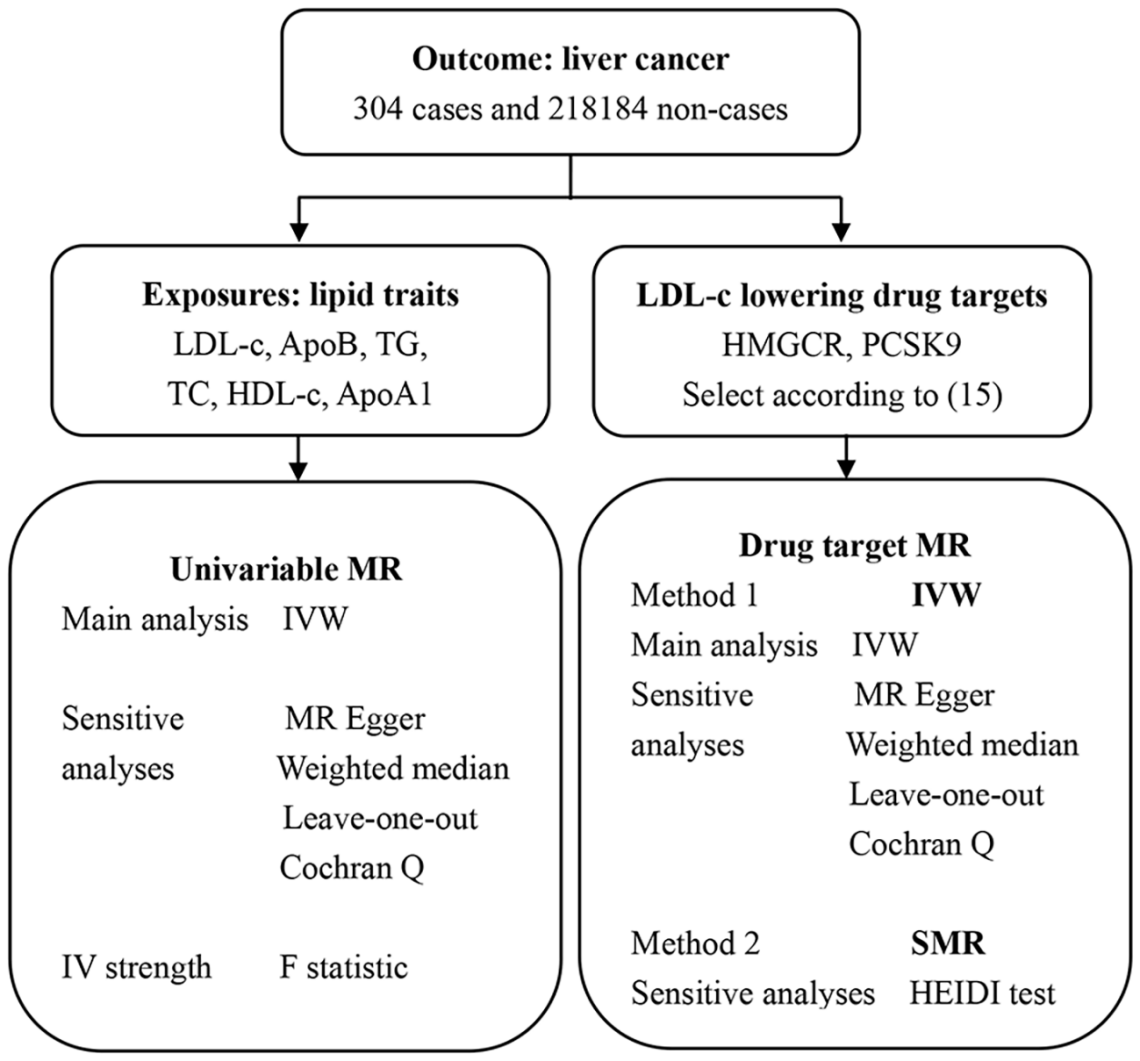
The design of the entire study.

In this study, we performed two types of MR analyses (**Figure 1**). Sequential univariable MR analysis was performed to assess the causal effects of individual lipid variables such as LDL-c, HDL-C, TG, TC, ApoB, and ApoA1, on the outcome (risk of liver cancer). Furthermore, DMR analysis was performed using the IVW and SMR methods to determine the causal effects of the SNPs in the target genes of LDL-c lowering drugs (HMGCR and PCSK9) on the outcomes.

### 2.2 GWAS data sources

The data reported in this study and the databases used in this study are shown in **Supplementary Material (Supplementary Table 1)**. The serum lipids data was extracted from the GWAS summary datasets using the MRC Integrative Epidemiology Unit (IEU) Open GWAS database (https://gwas.mrcieu.ac.uk/) at the University of Bristol. The genetic data for liver cancer samples was obtained from the meta-analysis of GWAS and included 304 liver cancer cases and 218,184 controls (https://gwas.mrcieu.ac.uk/). Liver cancer samples included cases of hepatocellular carcinoma, cholangiocarcinoma, hepatic adenoma, hepatic hemangioma, and liver metastases. For this study, we selected a genetically representative dataset that exhibited strong representativeness. Based on the original research ethics approval for GWAS, the informed consent of patients and other ethical requirements were waived for this study.

### 2.3 Selection criteria for the instrumental variables

Single nucleotide polymorphisms (SNPs) for liver cancer we identified from the FinnGen database and analyzed as the IVs. For the univariable MR analyses, SNPs that showed strong association with liver cancer were selected as instrumental variables (IVs) using the genome-wide significance (WGS) threshold P value of <5 × 10^−8^. These SNPs were further screened with criteria: linkage disequilibrium R^2^ value < 0.001 and distance between adjacent SNPs is less than 10 Mb. Palindromic IVs with intermediate allele frequencies were excluded. Furthermore, a threshold F-statistic value > 10 was applied to exclude genetic variations as potential IVs. For the DMR analysis, eQTLs for the drugs target gene (HMGCR and PCSK9) were used as proxies for exposure to each drug reducing the LDL-c levels. The summary-level data for the eQTLs was obtained from the eQTLGen Consortium (https://www.eqtlgen.org/) or the SMR Yanglab database (https://yanglab.westlake.edu.cn/software/smr/). Then, common eQTLs were identified for the SNPs with a minor allele frequency (MAF) of > 1% and significant association (p < 5.0 × 10^−8^) with the serum expression levels of HMGCR or PCSK9. Genetic instruments were generated using only the cis-eQTLs, which were defined as eQTLs located within a range of 100 kb range on either side of the encoded gene. The summary-based MR (SMR) method was used to confirm the association between eQTLs and the GWAS-based instruments variables.

### 2.4 MR analyses

In the univariable MR analysis, the IVW method was used to investigate the causal relationship between a 1 -standard deviation (SD) increase in the genetically predicted risk and the outcome. MR Egger method was used to determine horizontal pleiotropy in the univariable MR analyses. Cochran’s Q statistics and the MR-Egger tests were employed used to estimate heterogeneity and directional pleiotropy. The MR-PRESSO approach was used to remove any potential “outliers” among the original set of SNPs and a set of SNPs that contributed significantly to the observed high heterogeneity were identified to ensure high reliability. For the DMR analysis by the IVW method, heterogeneity was tested using the Cochran Q test and p < 0.05 was used as a threshold to indicate heterogeneity. For the SNPs that were used as instrument variables horizontal pleiotropy was assessed using the MR-Egger regression analysis and the MR-PRESSO analysis (global test > 0.05), as previously described by Huang W et al. (15). The intercept term in MR Egger regression was used in this study as an indicator of directional horizontal pleiotropy and, p < 0.05 was used as the threshold parameter to identify horizontal pleiotropy. Furthermore, SMR software was used to perform the heterogeneity in dependent instruments (HEIDI) test (p_ HEIDI > 0.01) to determine whether the observed association between gene expression levels and the outcome were caused by a linkage.

## 3 Results

### 3.1 Causal effects of serum lipid indicators on liver cancer

**Figure 2:**
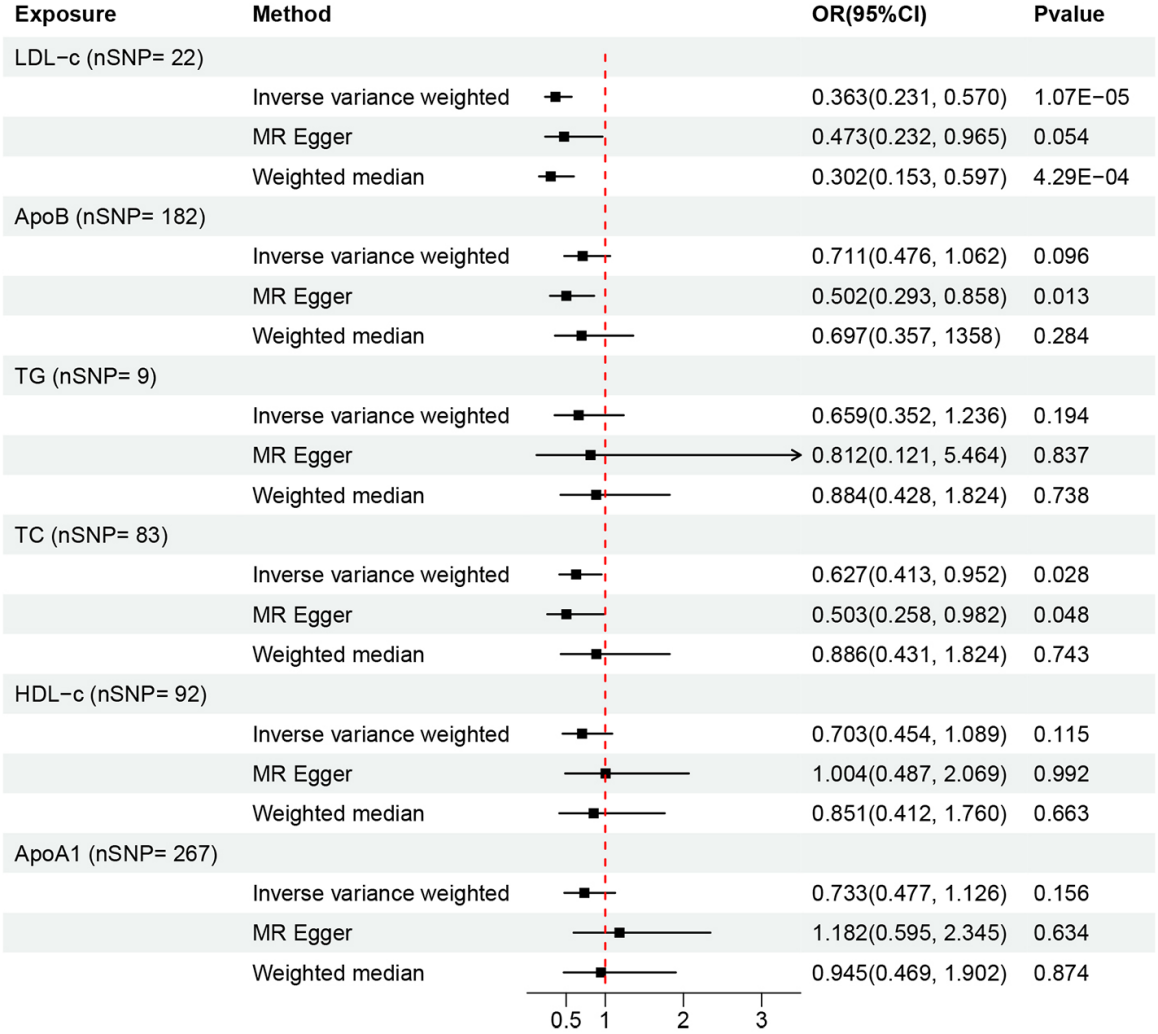
Summary of the univariable MR analysis results for the association between lipid-related factors and the risk of liver cancer using the IVW, MR Egger, and Weighted median methods. nSNP, number of SNPs.

In the univariable MR analysis, all SNPs of serum lipid indicators and liver cancer that were analyzed by F statistics were greater than 10. The harmonize function was used to adjust the results of serum lipid indicators and liver cancer, aligning the effect allele of IVs with the action=2 threshold. The number of SNPs (nSNP) in the causal relationship between serum lipid indicators and liver cancer are as follows: 22 for LDL-c, 182 for ApoB, 9 for TG, 83 for TC, 92 for HDL-c, and 267 for ApoA1 (**Figure 2**, **Supplementary Tables 2-7**). The SNPs (rs188247550, rs72999033) of TG and liver cancer were excluded, as their MR-PRESSO analysis P-values were 0.0011 and 0.0495, respectively (both <0.05). Therefore, after aforementioned 2 SNPs were removed, only 9 were retained. The univariable MR analysis was performed using the IVW, MR Egger, and Weighted median methods. This resulted in a simultaneous analysis of sensitivity and horizontal pleiotropy for the SNPs. MR-PRESSO analysis was used to remove the outliers with high heterogeneity. Global test was performed to verify the stability of the outcome. The relationship between individual lipid-related factors and liver cancer was analyzed using the IVW method and significant association was observed between liver cancer and serum levels of LDL-c and TC. An increase of 1-SD in the serum LDL-c levels was associated with decreased risk of liver cancer [odds ratio (OR) = 0.363; 95% CI= 0.231 - 0.570; p= 1.070E-5] (**Supplementary Figures 1-3**). An increase of 1-SD in serum TC levels was associated with decreased risk of liver cancer [OR = 0.627; (95% CI =, 0.413 - 0.952; p = 0.028] (**Supplementary Figures 4-6**). The stability of the results was assessed using the MR-Egger method. Liver cancer showed negative correlation with the levels of APOB (OR = 0.502, 95% CI = 0.293 - 0.858)and TC (OR = 0.503, 95% CI = 0.258 - 0.982). Weighted median analysis confirmed the negative correlation between serum LDL-c levels and the risk of liver cancer (OR = 0.302, 95% CI = 0.153 - 0.597). However, we did not observe any significant association between the serum levels of TG, HDL-c, and ApoA1and liver cancer (**Figure 2**).

### 3.2 Gene-specific analyses of the causal effects of the lipid-lowering drugs on liver cancer

**Figure 3:**
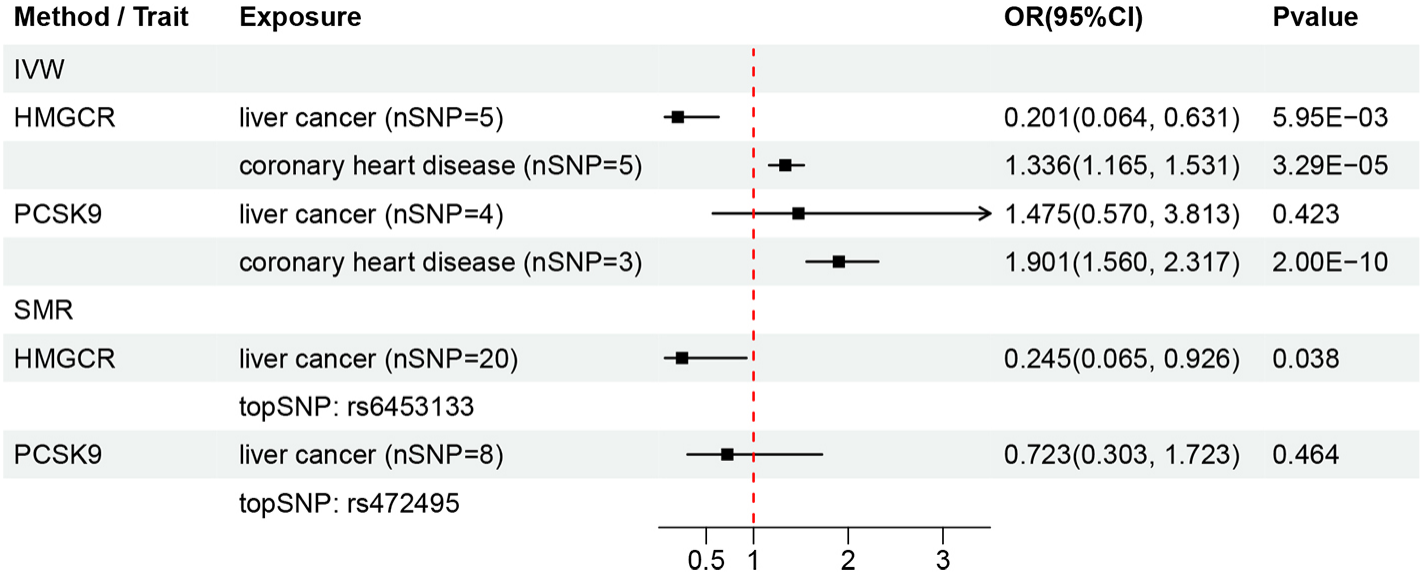
Summary of results from the DMR analysis. nSNP, number of SNPs. topSNP, the top ranked SNP.

There are information for the genetic instruments used in the DMR and associations for gene regions targeted by LDL-c lowering drugs (**Table 1**, **Figure 2**). IVW-MR analysis showed an inverse relationship between the serum LDL-c levels mediated by HMGCR and the risk of liver cancer (OR = 0.201, 95%CI = 0.064 - 0.631; p = 5.95E-03) (**Supplementary Figures 7-9**). However, the IVW-DMR analysis did not show any significant association between serum LDL-c levels mediated by PCSK9 and the risk of liver cancer (**Supplementary Figures 10-12**). In the SMR analysis, serum LDL-c levels mediated by HMGCR showed negative correlation with the risk of liver cancer (OR = 0.245, 95%CI = 0.065 - 0.926; p = 0.038) (**Figure 3**). Furthermore, SMR analysis did not show any significant association between the serum LDL-c levels mediated by PCSK9 and the risk of liver cancer. The intercept terms in the MR-Egger regression and MR-PRESSO analyses did not show any evidence of horizontal pleiotropy (all p > 0.05). The selected genetic instruments were validated by analyzing the correlations between drug-related SNPs and the coronary heart disease as the positive control (Pvalues for HMGCR and PCSK9 were 3.291E-05 and 2.000E-10, respectively) (**Figure 3**, **Supplementary Figures 13-18**). The HEIDI test results showed that all the observed associations were true and were not related to genetic linkage (p _ HEIDI values for HMGCR and PCSK9 were 0.550 and 0.922, respectively, both > 0.01).

**Table 1:** Summary of the genetic instruments used for the DMR analysis.

Exposures	Genetic variants (eQTLs) from the eQTLGen Consortium or SMR Yanglab based on mRNA expression levels	Genetic variants related to the serum LDL-c levels
HMGCR inhibitors	921 common cis-eQTLs (MAF>1%) in blood for the HMGCR gene (p < 5.0 × 10^−8^) with rs6453133 as the top ranked SNP.	5 common SNPs (MAF >1%) with low linkage disequilibrium (R^2^ < 0.40)for the association with the serum LDL-c levels (p < 5.0 × 10^−8^) and located within ± 100 kb from the HMGCR genetic locus.
PCSK9 inhibitors	24 common cis-eQTLs (MAF >1%) in blood for the PCSK9 gene (p < 5.0 ×10^−8^) with rs472495 as the top ranked SNP.	4 common SNPs (MAF > 1%) with low linkage disequilibrium (R^2^ < 0.40), for the association with the serum LDL-c (p < 5.0 × 10^−8^) and located within ± 100 kb from the PCSK9 genetic locus.

## 4 Discussion

The liver is an important site of lipid metabolism. Therefore, aberrant changes in lipid metabolism, including cholesterol metabolism can influence the development of liver tumors (16). Therefore, different types of in lipid molecules and enzymes related with lipid metabolism may serve as potential prediction and diagnostic biomarkers of liver diseases (17). Aberrant lipid metabolism causes metabolic syndrome, including non-alcoholic fatty liver disease (NAFLD) (18, 19). Drugs that reduce aberrant lipid levels have been shown to delay the symptoms of NAFLD, but their effects on the risk of liver cancer at the genetic level are unknown. Therefore, we performed univariate MR analysis to determine the causal effects of changes in the serum levels of various lipid factors on the risk of liver cancer. Subsequently, we performed a predictive DMR analysis to determine the association of genes targeted by the lipid-lowering drugs with liver cancer.

The present univariable MR study demonstrated that low serum levels of LDL-c and TC were associated with an increased risk of liver cancer. These findings suggested that high serum LDL-c and TC levels protected against liver cancer. However, our data did not show any significant correlation between serum HDL-c, TG, TC, ApoB, and ApoA1 levels and the risk of liver cancer. The current study did not show further evidence to validate the causal relationship between serum LDL-c levels and the risk of liver cancer. Furthermore, we performed a causal MR analysis to determine the relationship between serum LDL-c levels and liver cancer based on the genes that regulate LDL-c levels and the drugs that target LDL-c metabolism. Ahn J et al. performed a follow-up study of an European cohort and high serum levels of TC correlated with a reduced risk of liver cancer (RR = 0.66, 95% CI = 0.43 – 1.01, P trend = 0.007) (20). Ma X et al. performed a prospective cohort study of an Asian population and reported that reduced TC levels were associated with a higher risk of liver cancer [univariate model: 0.72 (0.66 - 0.78); multivariate model: 0.78 (0.72 – 0.86)] (10). Our data concurred with the findings of these two studies. Our study also analyzed the causal relationship between serum LDL-c levels and liver cancer based on the genetic prediction. Previous studies have shown significant association of reduced serum levels of LDL-c and TC with cancer. A previous observational study demonstrated that lower serum LDL-c levels (< 130mg/dL) and TC levels (< 200mg/dL) were associated with prostate cancer (21). Zhang X et al. reported that the LDL-c levels in colorectal cancer patients were lower than those in the healthy subjects (22). However, the causal relationship between LDL-c levels and colorectal cancer is still unclear. Furthermore, serum TC levels show an inverse association with the risk of breast cancer risk [HR (1mmol/L increment) = 0.83, 95% CI: 0.69 - 0.99; P = 0.04] (23). Another study reported an inverse association between breast cancer risk and increasing serum TC levels (OR =0.46, 95% CI = 0.25 - 0.85) and serum LDL-c levels (OR = 0.41, 95% CI = 0.21 - 0.81) (24). Therefore, our findings suggested an inverse correlation between serum LDL-c and TC levels and the risk of liver cancer.

The causal relationship between lipid-lowering drugs and liver cancer remains inconclusive. Population-based studies have suggested that the use of statins may reduce the risk of hepatocellular carcinoma (HCC). However, correlation between statin use and intrahepatic bile duct carcinoma (IBDC) has not been reported. A large cohort randomized trial demonstrated that reduction of LDL-c levels by statins was associated with an increased risk of cancer development (25). The inhibitors of PCSK9 reduce serum LDL-c levels and show a correlation with the occurrence and progression of liver cancer (26). Our study investigated the relationship between liver cancer and the effects of inhibit HMGCR and PCSK9, respectively. However, the underlying mechanisms of these associations require further investigation. HMGCR and PCSK9 are targets of statins and PCSK9 inhibitors, respectively, for reducing LDL-c levels. In this study, we investigated the causal relationship between liver cancer and SNPs related to the HMGCR and PCSK9 genes, which regulate LDL-c levels. We also analyzed the side effects of LDL-c lowering drugs. The scientific validity of the SNPs was confirmed using the positive control. The results of both IVW and SMR methods showed an inverse association between serum LDL-c levels mediated by HMGCR and the risk of liver cancer. However, serum LDL-c levels mediated by PCKS9 did not show any significant association with the risk of liver cancer. Therefore, our study suggested a causal relationship between statins, which act as inhibitors of HMGCR, and liver cancer. HMGCR is the rate-limiting enzyme in the mevalonate pathway, which participates in cholesterol biosynthesis. Hepatic carcinoma cells with decreased expression of HMGCR showed reduced growth, migration, and colony formation ability (27).

Our study has several advantages of our study are as follows. Firstly, we demonstrated for the first time a causal relationship between serum LDL-cand TC levels and liver cancer by using GWAS data. Therefore, LDL-c and TC may be independent variables for predicting the risk of liver cancer. Secondly, we demonstrated that statins increased the risk of liver cancer based on the genetic data. Thirdly, we used various methods to validate the accuracy of MR results, including (1) estimating the F-statistic values to exclude weak IVs: (2) verifying the SNPs using a positive control group; and (3) using both the IVW and SMR methods to validate the accuracy of DMR results. Finally, DMR methodology uses genetic tools to replace drug exposure and minimizes confounding bias and reverse causality.

However, this study also has several limitations. Firstly, SNPs related to NPC1L1 and APOB, which are drug targets for reducing LDL-c and TC levels, respectively, were not found in the outcome gene database. Therefore, we could not analyze the association between liver cancer and the levels and activity of NPC1L1and APOB. Secondly, the sample size of liver cancer cases was small (n = 304). Thirdly, we cannot completely rule out confounding bias despite performing multiple sensitivity analyses to evaluate the results from the MR study. Fourthly, the data used in this study was based on the European population. Therefore, we should exercise caution when interpreting these findings for populations from other countries and continents.

## 5 Conclusions

Our data showed that reduced serum levels of low LDL-c and TC were associated with increased risk of liver cancer. Furthermore, reduced serum LDL-c levels mediated by HMGCR inhibition by statins were associated with reduced risk of liver cancer. Therefore, the use of statins for the treatment of subjects with lipid metabolic syndrome needs to be monitored for reducing the risk of liver cancer.

## Data availability statement

The original contributions presented in the study are included in the article/**Supplementary Material**. Further inquiries can be directed to the corresponding author.

## Author contributions

ZL wrote the manuscript and performed the quality assessment. ZL designed the project and performed the statistical analysis. ZZ and XT contributed to the revision of the manuscript and reviewed the results. Conceptualization: ZL and XT. Methodology: ZZ. Software: ZL. Validation: ZL. Formal analysis: ZL. Investigation: ZL. Resources: ZL. Data curation: ZL. Writing—original draft preparation: ZL. Writing—review and editing: ZL. Visualization: ZL. Supervision: PZ. Funding acquisition: PZ. All authors contributed to the article and approved the submitted version.
