# Supplementary material for: Lipids, cholesterols, statins and liver cancer: a Mendelian randomization study
Source: Front Oncol. 2023 Sep 6;13:1251873. doi: 10.3389/fonc.2023.1251873 (PMC10516570; doi:10.3389/fonc.2023.1251873)
Supplement: Supplementary file 1 [file DataSheet_1.pdf]

Supplementary Material

Supplementary Material

Supplementary Figures

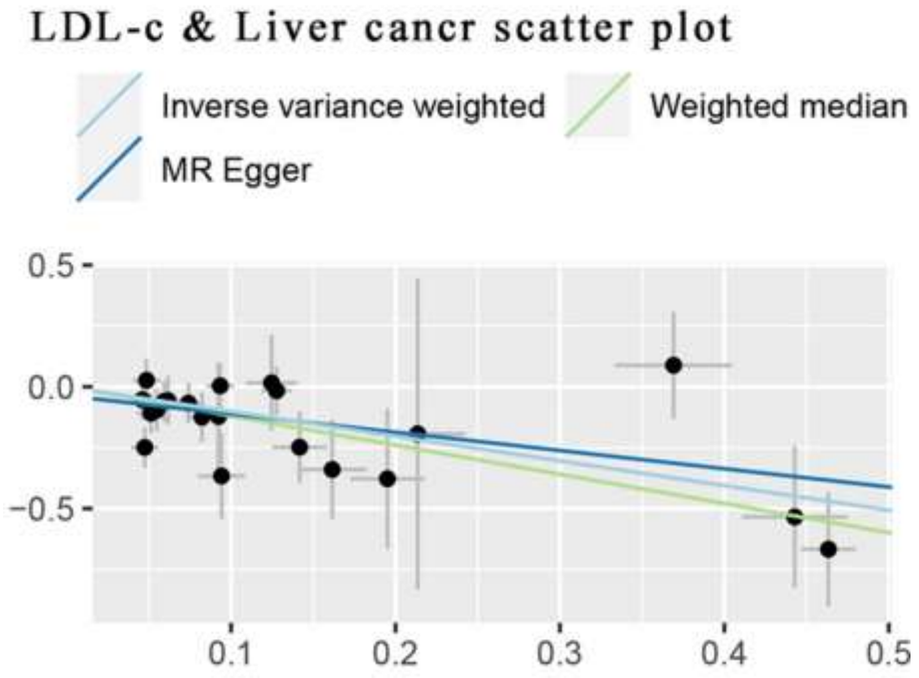

Supplemental Figure 1. Scatter plot of between serum LDL-c and Liver cancer TSMR analysis. LDL-c, low-density lipoprotein cholesterol; TSMR, two-sample Mendelian randomization.

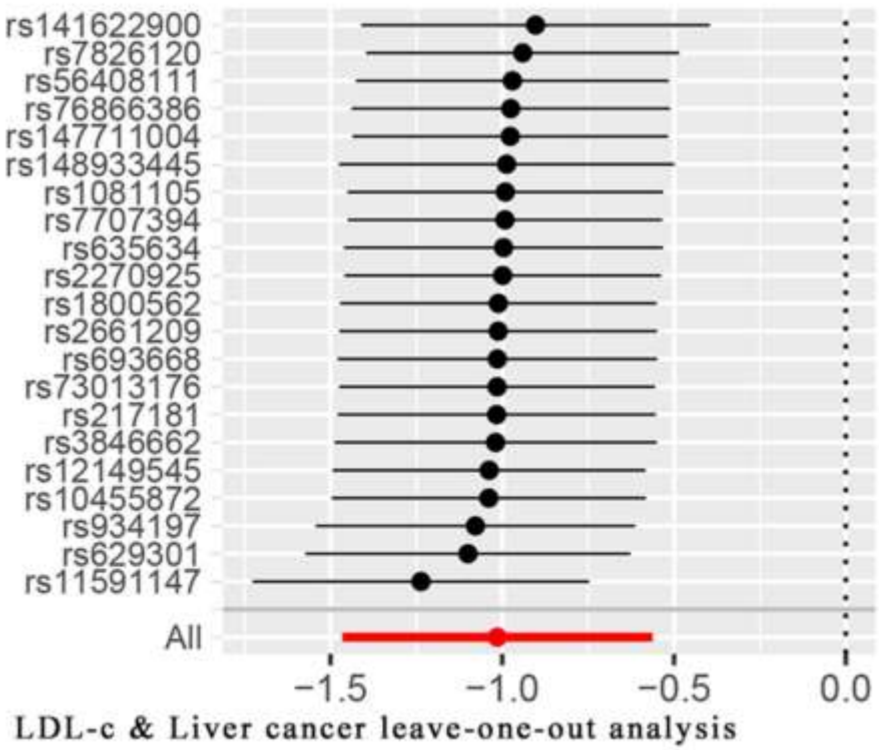

Supplemental Figure 2. Serum LDL-c and Liver cancer TSMR leave-one-out analysis. LDL-c, low-density lipoprotein cholesterol; TSMR, two-sample Mendelian randomization.

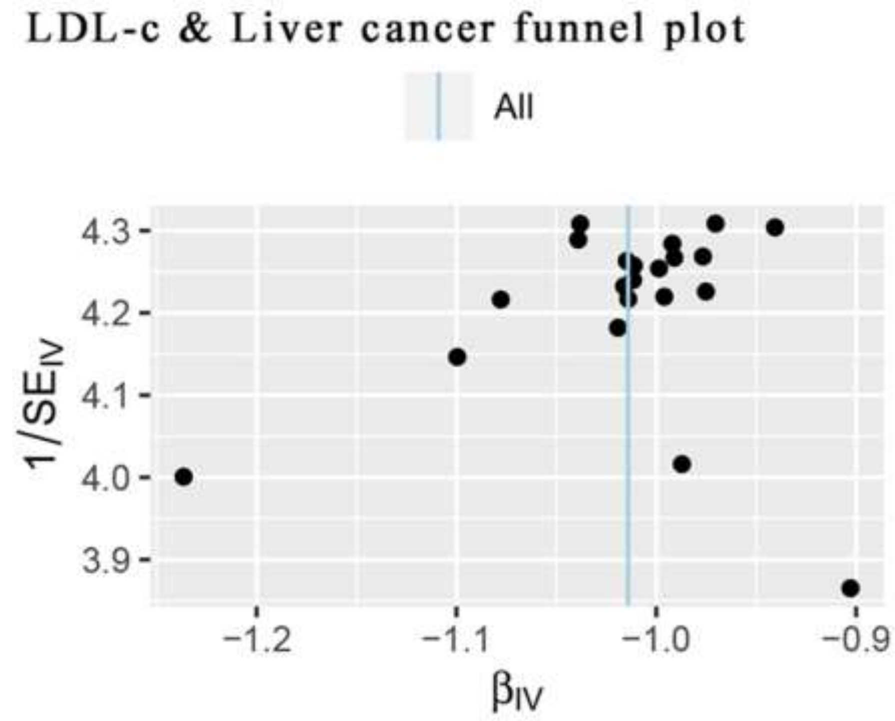

Supplemental Figure 3. Funnel plot of between serum LDL-c and Liver cancer TSMR analysis. LDL-c, low-density lipoprotein cholesterol; TSMR, two-sample Mendelian randomization.

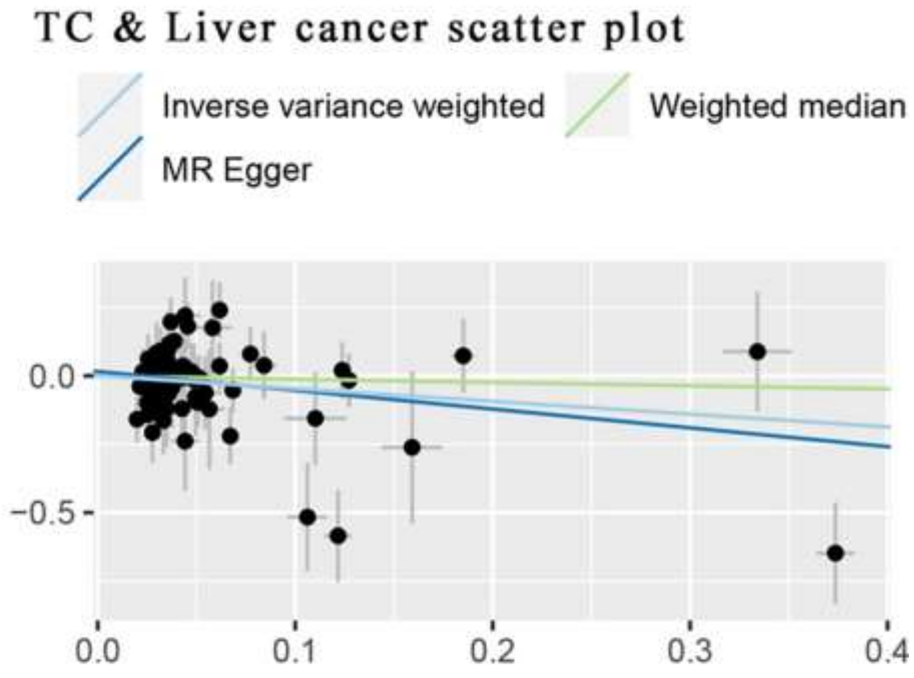

Supplemental Figure 4. Scatter plot of between serum TC and Liver cancer TSMR. TC, total cholesterol; TSMR, two-sample Mendelian randomization.

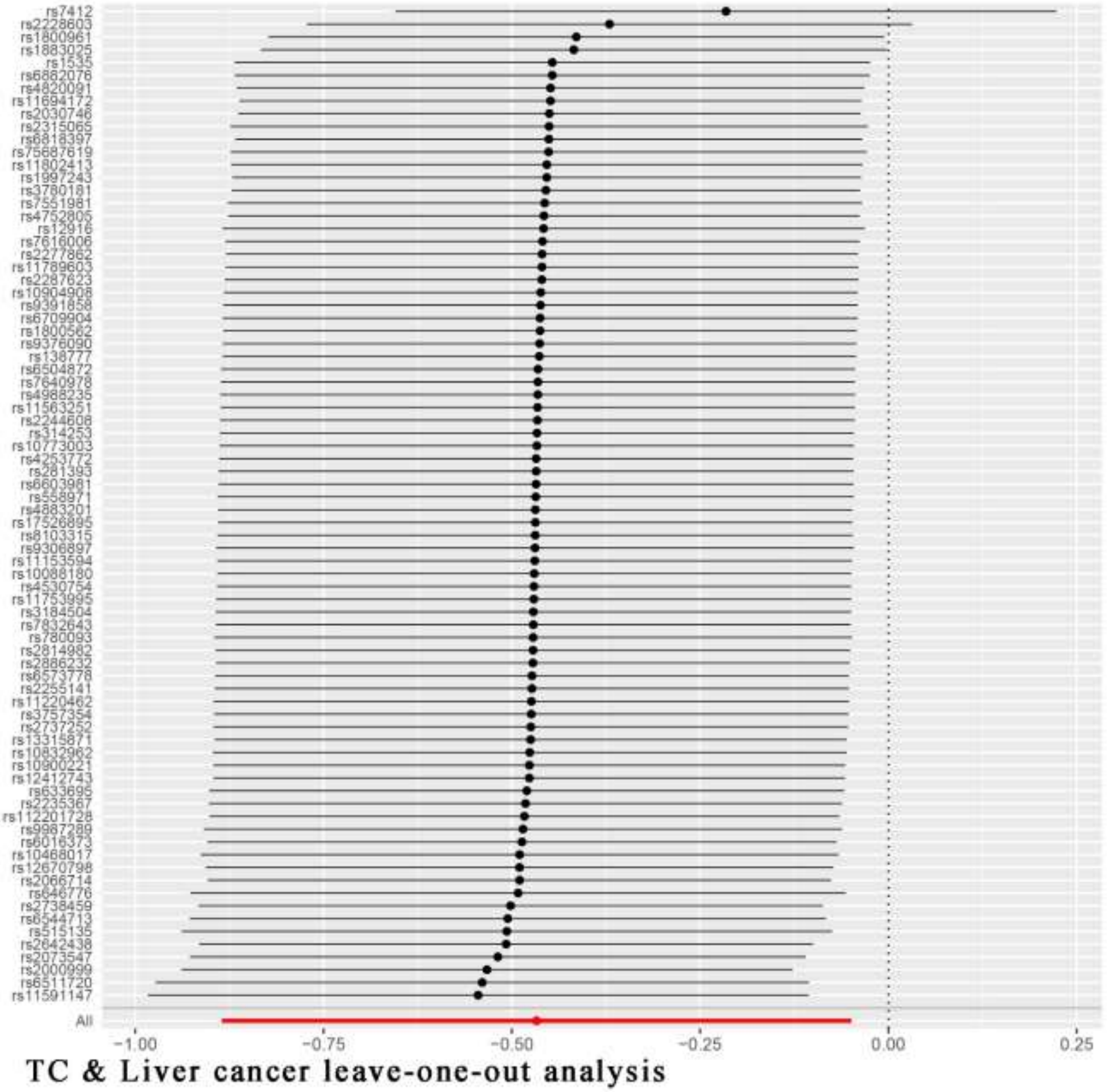

Supplemental Figure 5. Serum TC and Liver cancer TSMR leave-one-out analysis. TC, total cholesterol; TSMR, two-sample Mendelian randomization.

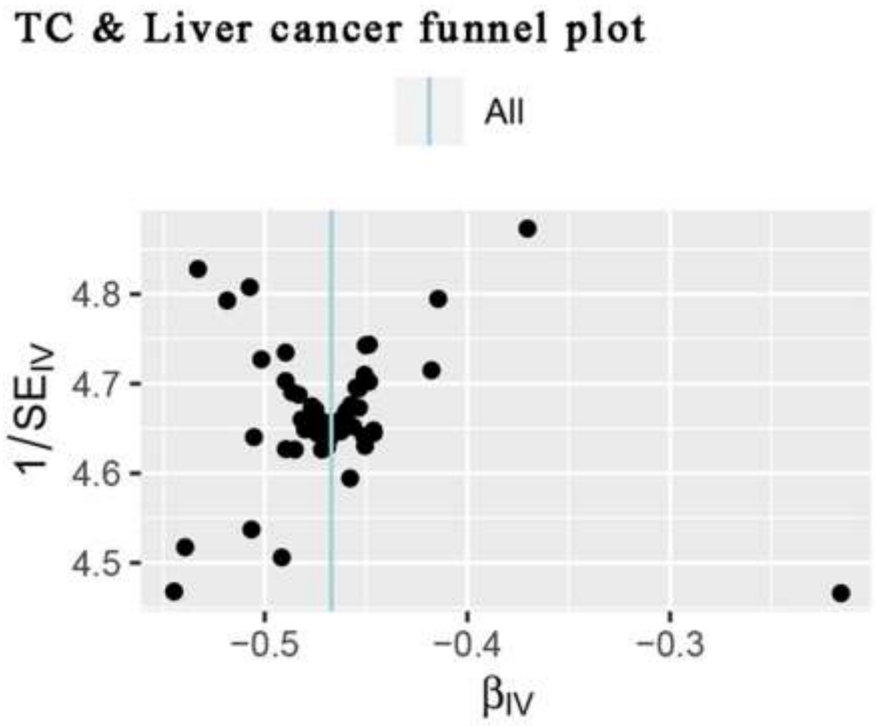

Supplemental Figure 6. Funnel plot of between serum TC and Liver cancer TSMR. TC, total cholesterol; TSMR, two-sample Mendelian randomization.

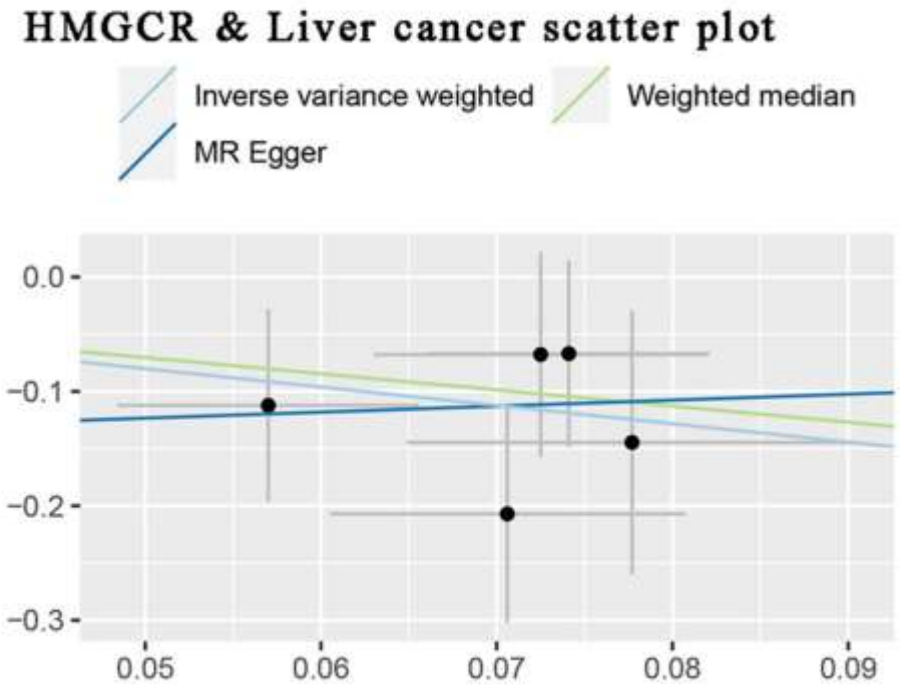

Supplemental Figure 7. Scatter plot of between HMGCR and Liver cancer DMR. DMR, drug target Mendelian randomization.

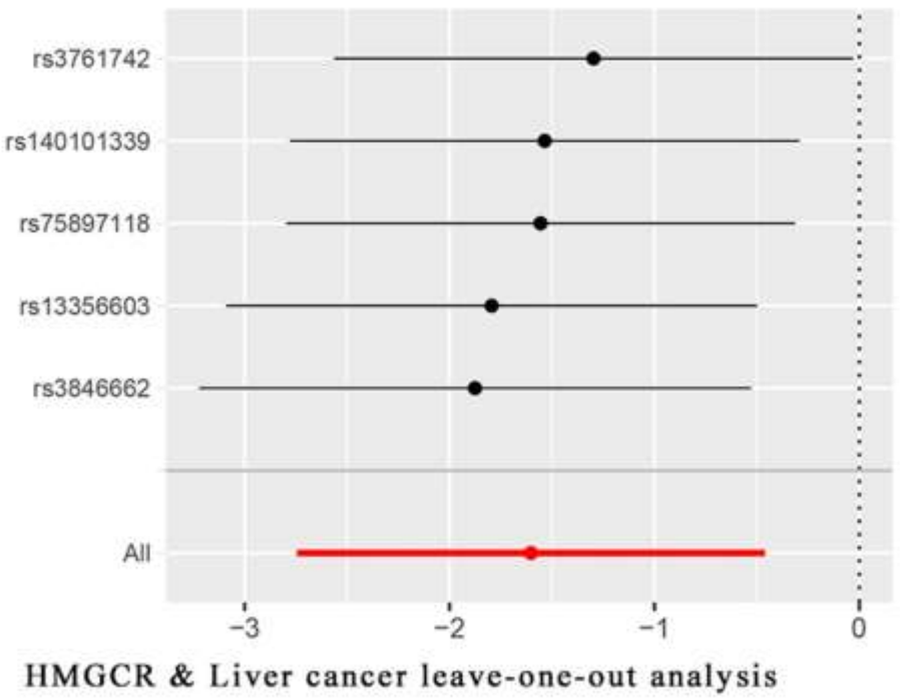

Supplemental Figure 8. HMGCR and Liver cancer DMR leave-one-out analysis. DMR, drug target Mendelian randomization.

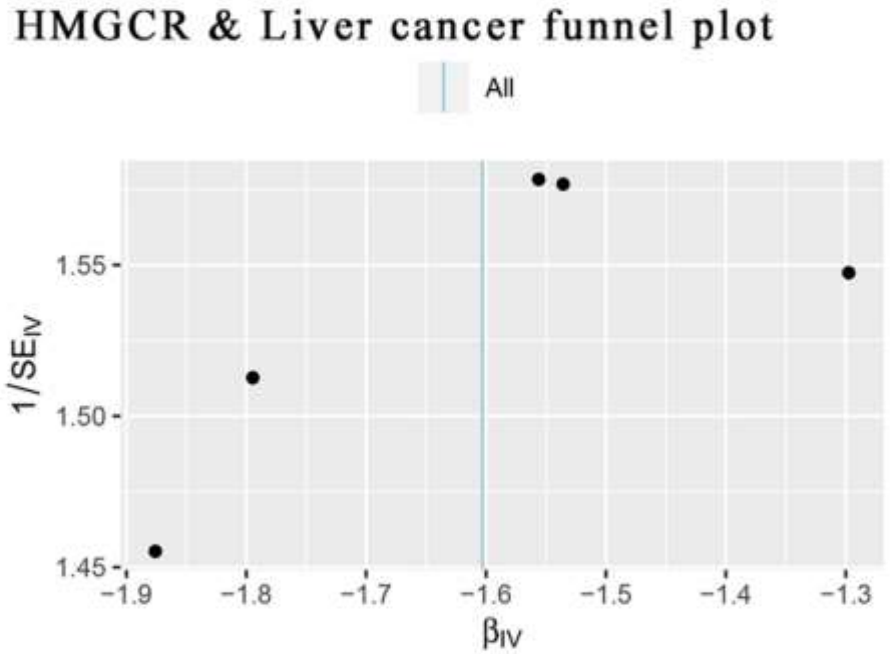

Supplemental Figure 9. Funnel plot of between HMGCR and Liver cancer DMR. DMR, drug target Mendelian randomization.

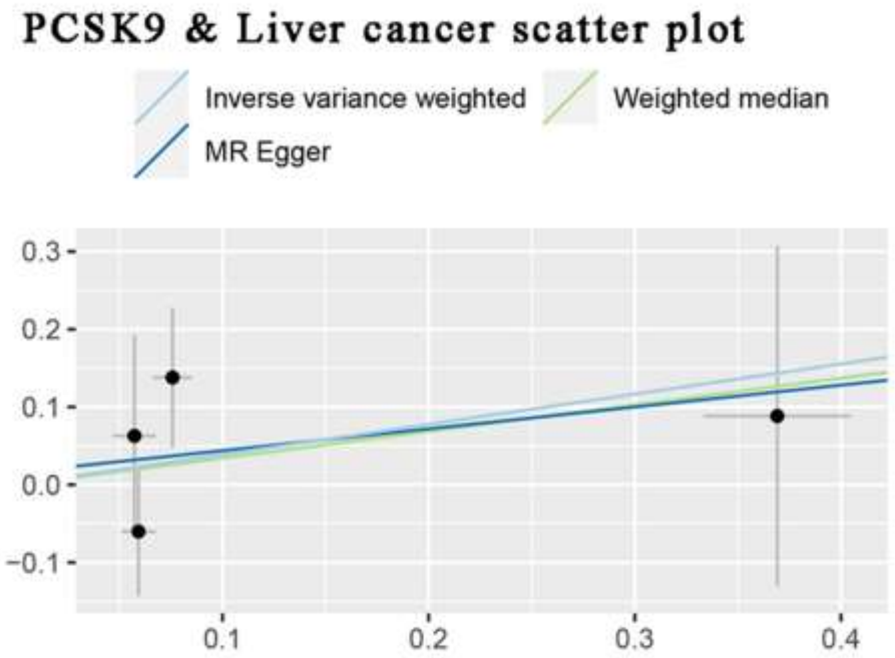

Supplemental Figure 10. Scatter plot of between PCSK9 and Liver cancer DMR. DMR, drug target Mendelian randomization.

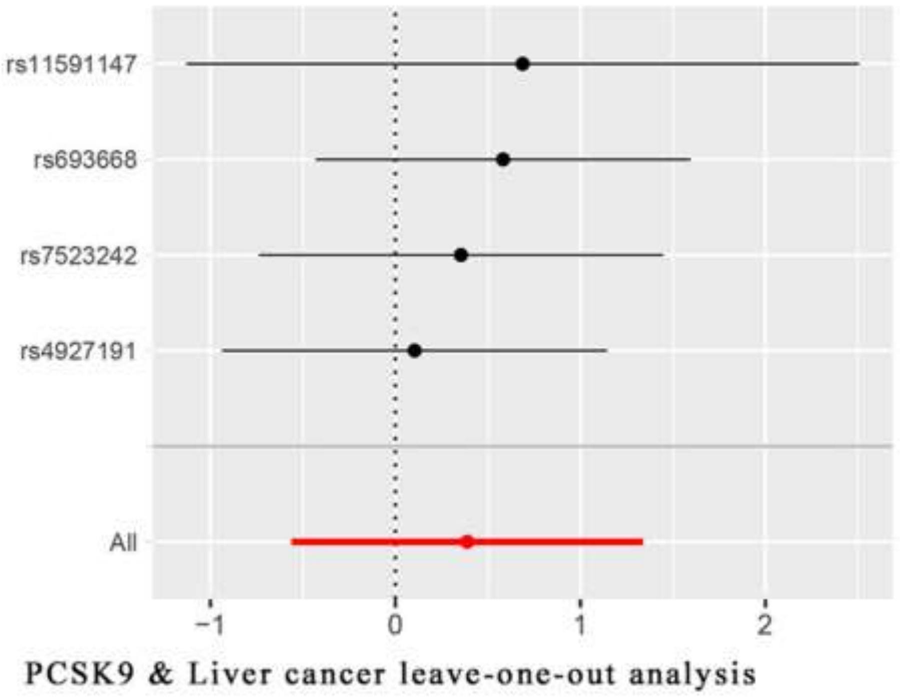

Supplemental Figure 11. PCSK9 and Liver cancer DMR leave-one-out analysis. DMR, drug target Mendelian randomization.

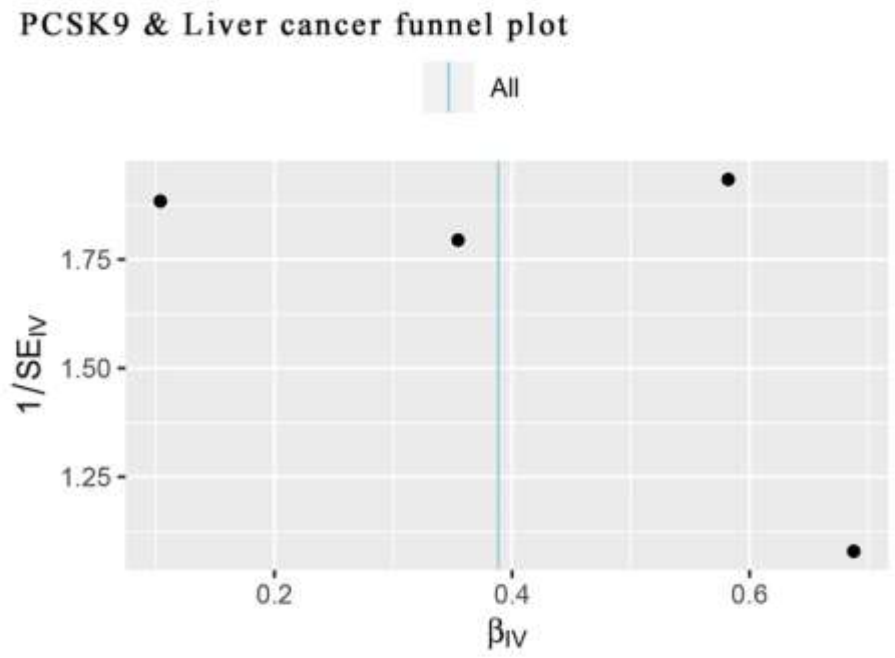

Supplemental Figure 12. Funnel plot of between PCSK9 and Liver cancer DMR. DMR, drug target Mendelian randomization.

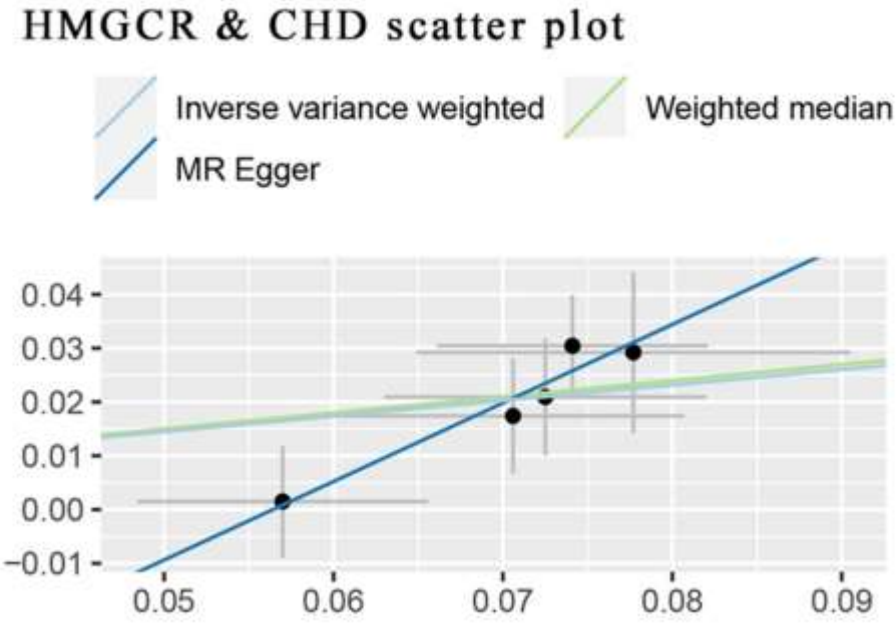

Supplemental Figure 13. Scatter plot of between HMGCR and Coronary Heart Disease DMR. DMR, drug target Mendelian randomization.

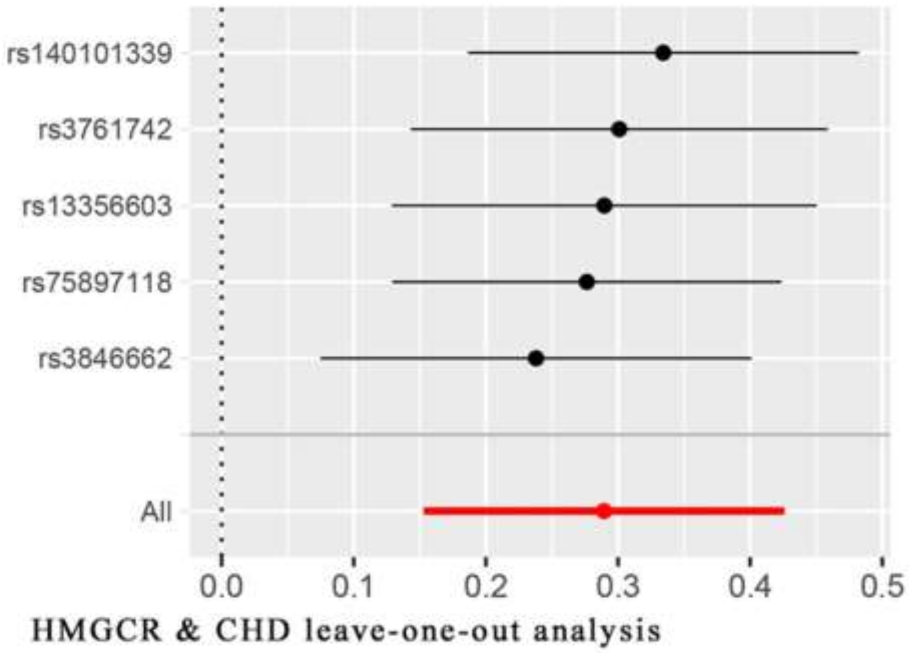

Supplemental Figure 14. HMGCR and Coronary Heart Disease DMR leave-one-out analysis. DMR, drug target Mendelian randomization.

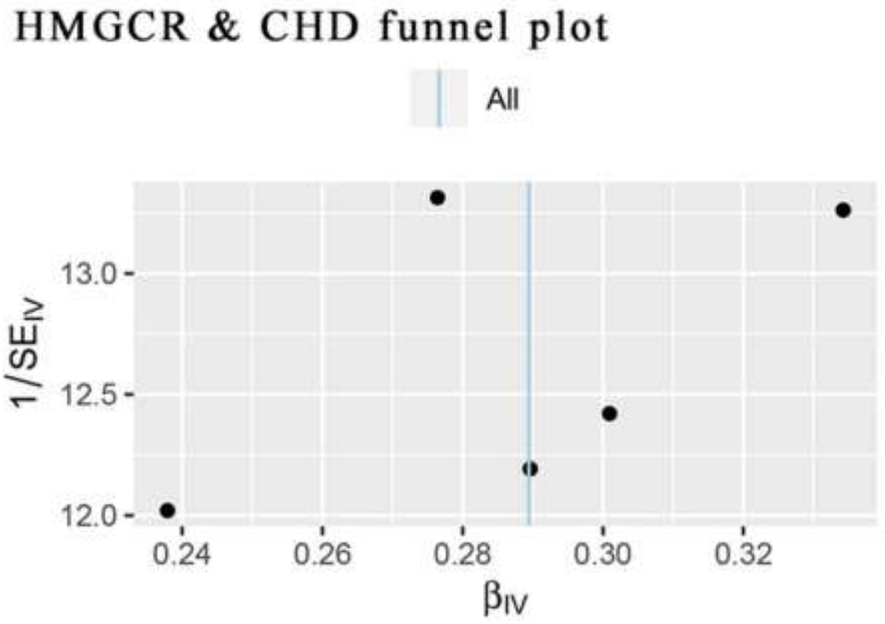

Supplemental Figure 15. Funnel plot of between HMGCR and Coronary Heart Disease DMR. DMR, drug target Mendelian randomization.

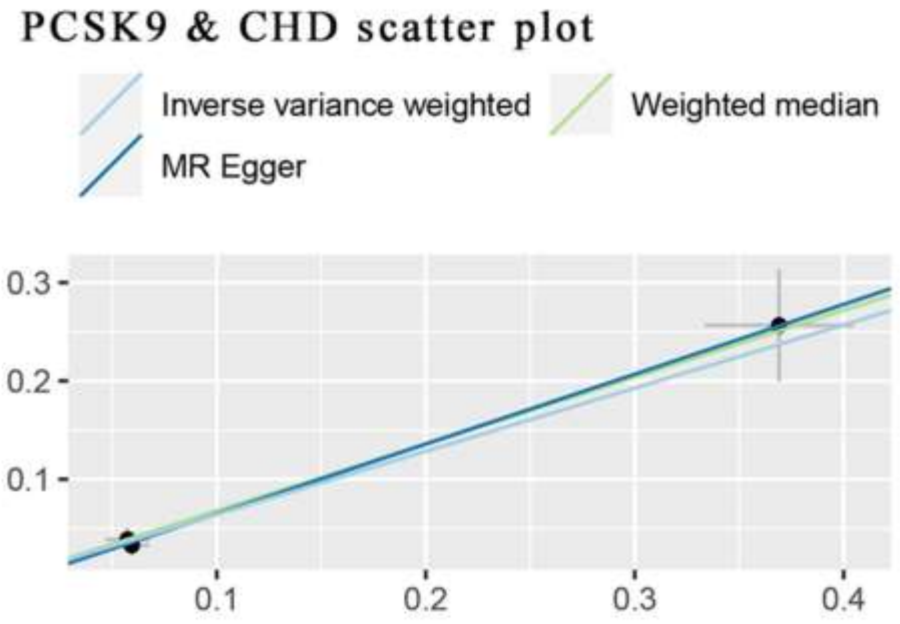

Supplemental Figure 16. Scatter plot of between PCSK9 and Coronary Heart Disease DMR. DMR, drug target Mendelian randomization.

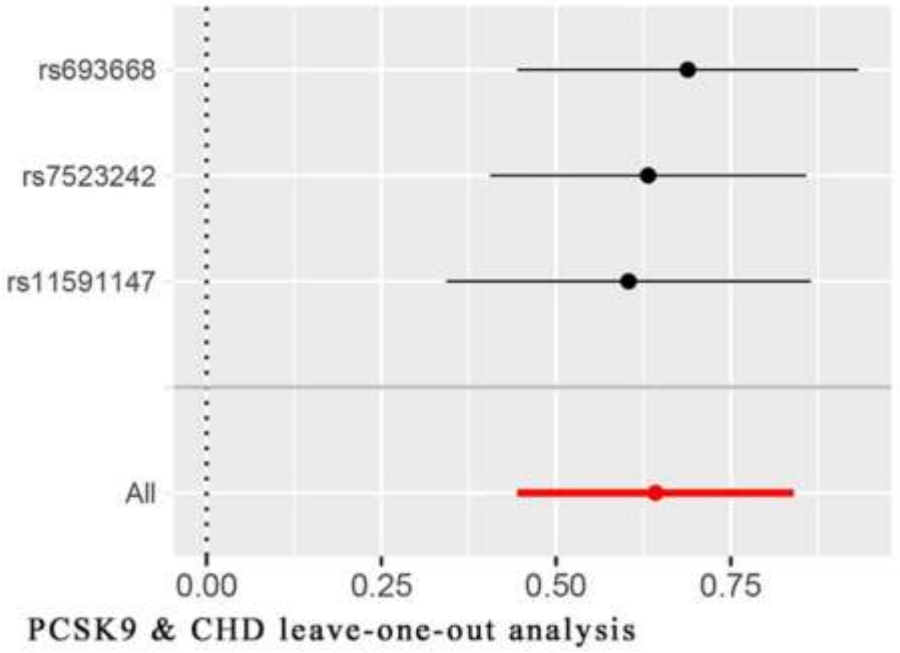

Supplemental Figure 17. PCSK9 and Coronary Heart Disease DMR leave-one-out analysis. DMR, drug target Mendelian randomization.

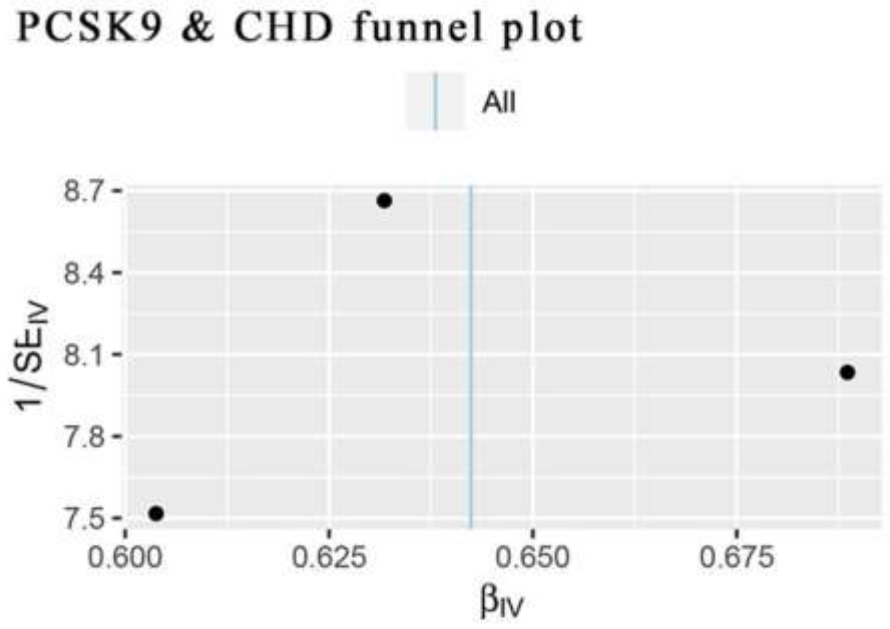

Supplemental Figure 18. Funnel plot of between PCSK9 and Coronary Heart Disease DMR. DMR, drug target Mendelian randomization.
